# Supplementary figures and images for: Immunophenotyping Reveals Longitudinal Changes in Circulating Immune Cells During Radium-223 Therapy in Patients With Metastatic Castration-Resistant Prostate Cancer
Source: Front Oncol. 2021 May 18;11:667658. doi: 10.3389/fonc.2021.667658 (PMC8167220; doi:10.3389/fonc.2021.667658)

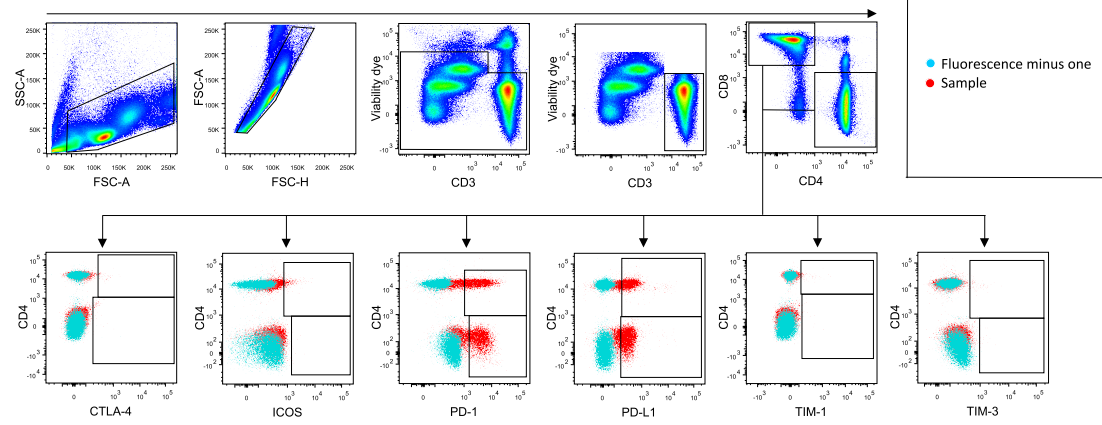

T cell panel

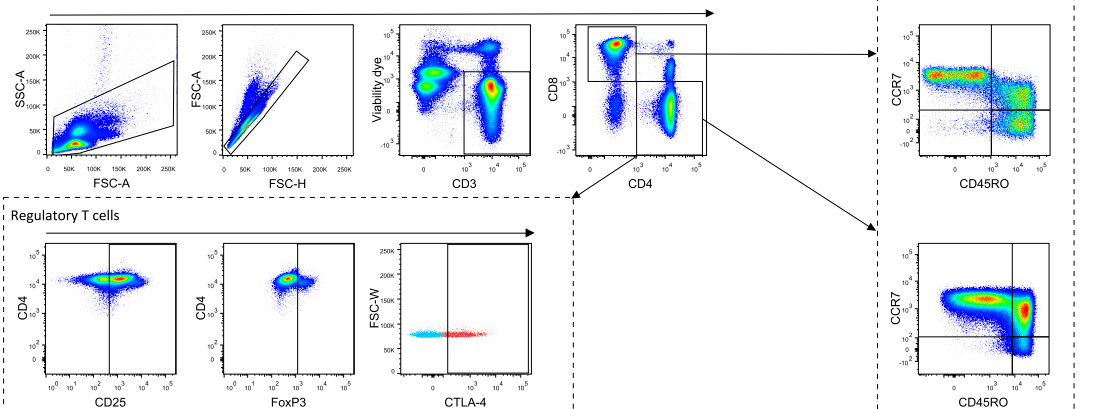

MDSC panel

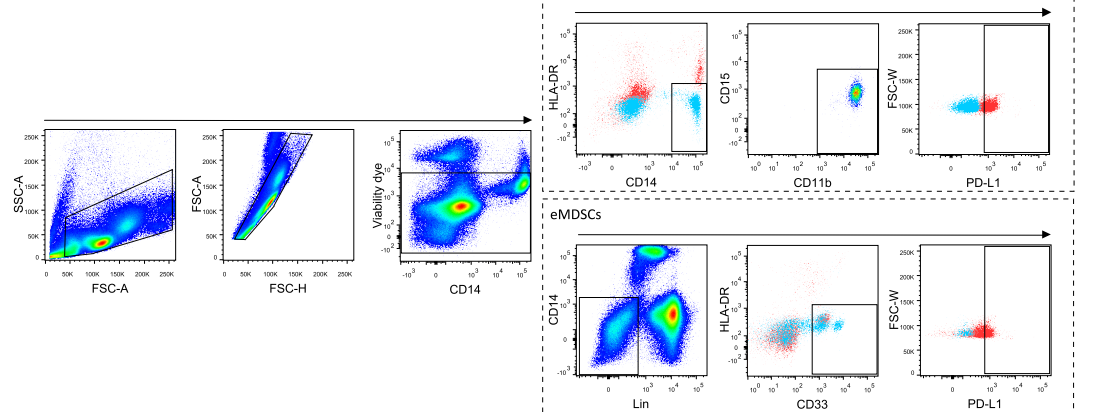

Supplement: Supplementary file 1 [file DataSheet_1.pdf]

Patient

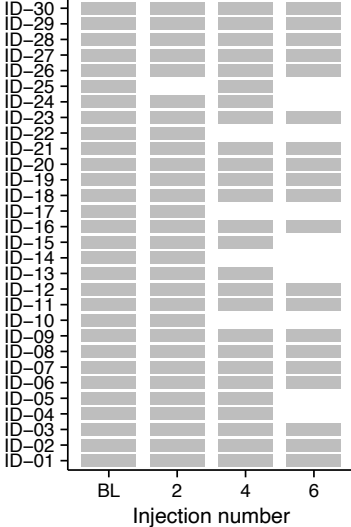

Supplement: Supplementary file 2 [file DataSheet_2.pdf]

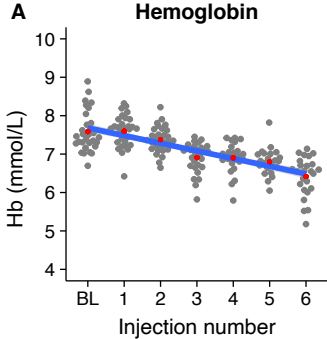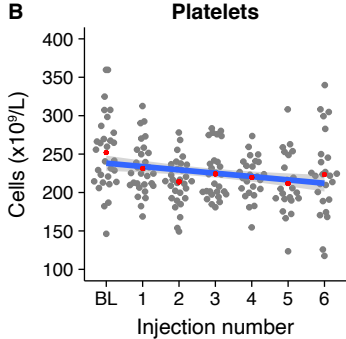

Supplement: Supplementary file 3 [file DataSheet_3.pdf]

$\Delta$ MFI

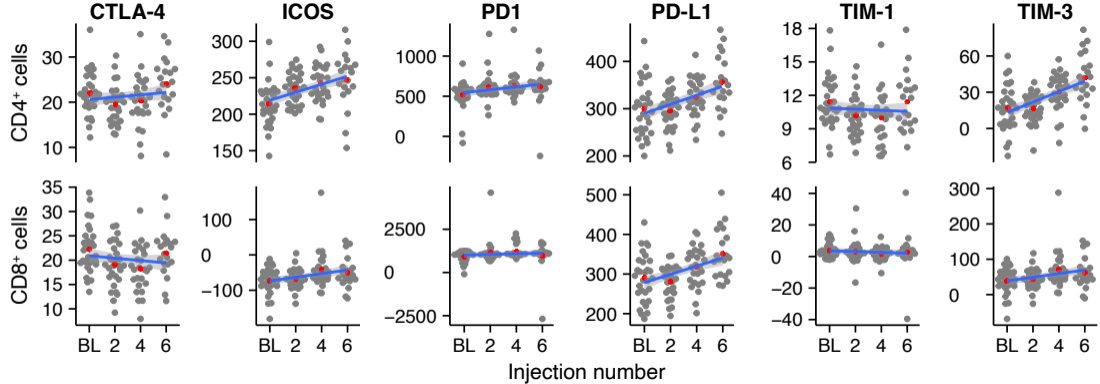

Supplement: Supplementary file 4 [file DataSheet_4.pdf]

# Checkpoint molecules

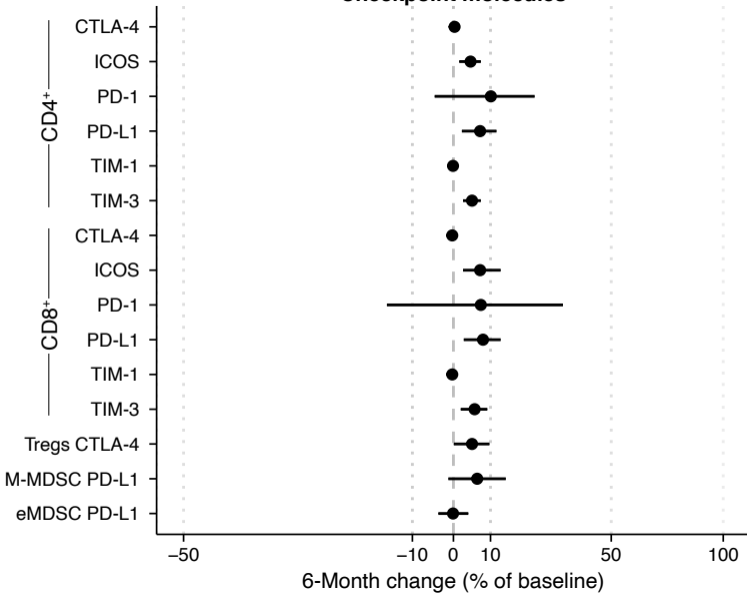

Supplement: Supplementary file 5 [file DataSheet_5.pdf]

Patient

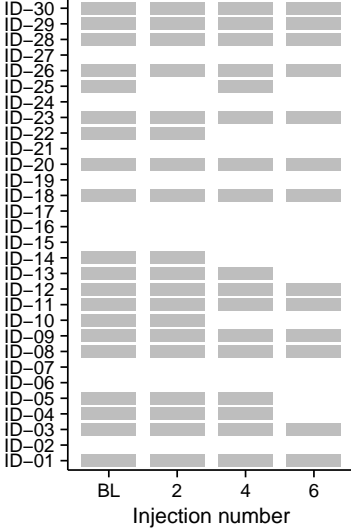

Supplement: Supplementary file 6 [file DataSheet_6.pdf]

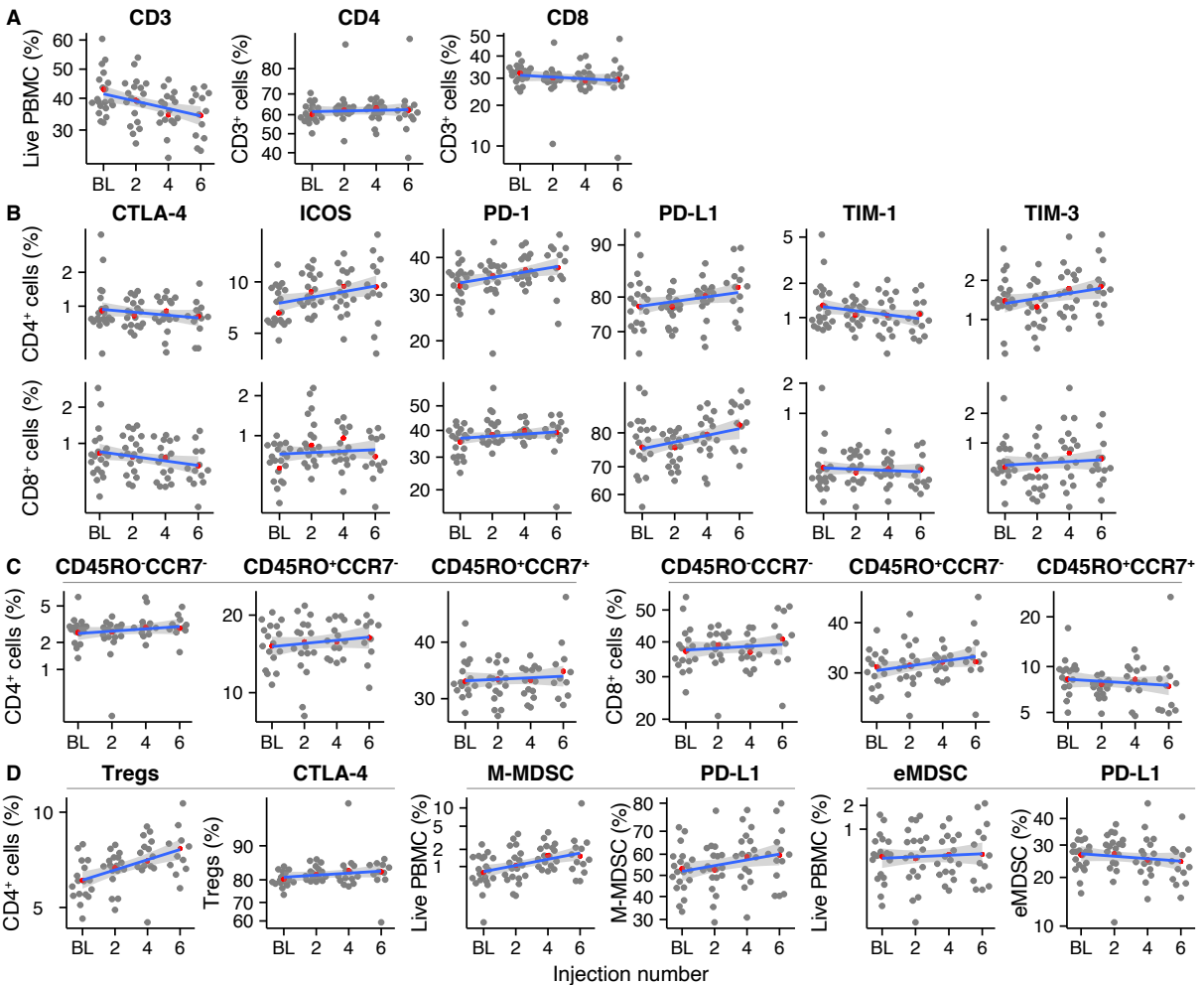

Supplement: Supplementary file 7 [file DataSheet_7.pdf]

**A****Main T cells**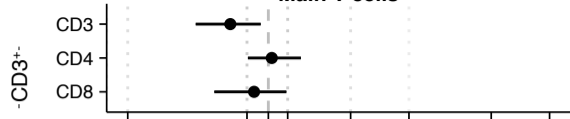**B****Memory/effector T cells**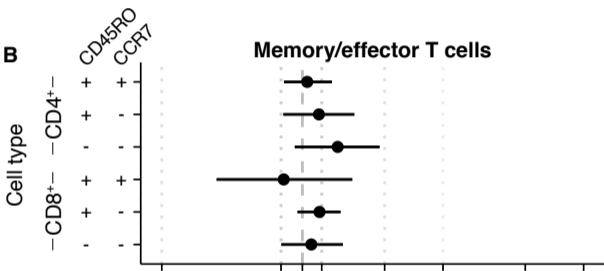**C****Immunosuppressive cells**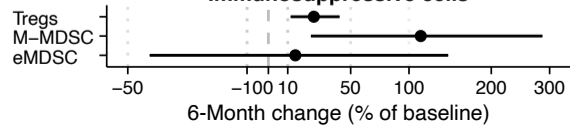**D****Checkpoint molecules**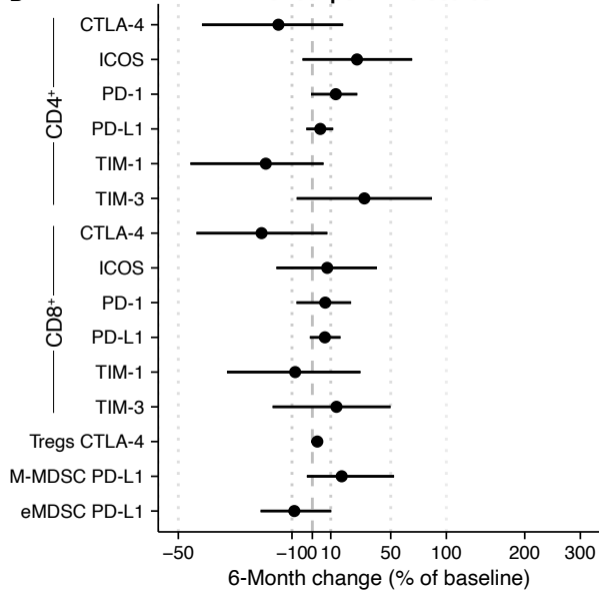

Supplement: Supplementary file 8 [file DataSheet_8.pdf]
